# Prevalence and Risk Factors of CoronaVac Side Effects: An Independent Cross-Sectional Study among Healthcare Workers in Turkey

**DOI:** 10.3390/jcm10122629

**Published:** 2021-06-15

**Authors:** Abanoub Riad, Derya Sağıroğlu, Batuhan Üstün, Andrea Pokorná, Jitka Klugarová, Sameh Attia, Miloslav Klugar

**Affiliations:** 1 Czech National Centre for Evidence-Based Healthcare and Knowledge Translation (Cochrane Czech Republic, Czech EBHC, JBI Centre of Excellence, Masaryk University GRADE Centre), Institute of Biostatistics and Analyses, Faculty of Medicine, Masaryk University, Kamenice 5, 625 00 Brno, Czech Republic; apokorna@med.muni.cz (A.P.); klugarova@med.muni.cz (J.K.); klugar@med.muni.cz (M.K.); 2 Department of Public Health, Faculty of Medicine, Masaryk University, Kamenice 5, 625 00 Brno, Czech Republic; 3 Department of Prosthetic Dentistry, Faculty of Dentistry, Yeditepe University, Bagdat Caddesi No. 238, Goztepe, Kadikoy, Istanbul 34728, Turkey; derya.sagiroglu1@std.yeditepe.edu.tr; 4 Department of Gynaecology and Obstetrics, Faculty of Medicine, Namık Kemal University, Namık Kemal Kampüs Caddesi No. 1, Merkez, Tekirdağ 59030, Turkey; bustun@nku.edu.tr; 5 Department of Nursing and Midwifery, Faculty of Medicine, Masaryk University, Kamenice 5, 625 00 Brno, Czech Republic; 6 Department of Oral and Maxillofacial Surgery, Justus-Liebig-University, Klinikstrasse 33, 35392 Giessen, Germany; sameh.attia@dentist.med.uni-giessen.de

**Keywords:** CoronaVac, COVID-19, drug-related side effects and adverse reactions, health personnel, mass vaccination, prevalence, Turkey

## Abstract

Background: COVID-19 vaccine hesitancy is a serious threat to mass vaccination strategies that need to be accelerated currently in order to achieve a substantial level of community immunity. Independent (non-sponsored) studies have a great potential to enhance public confidence in vaccines and accelerate their uptake process. Methods: A nationwide cross-sectional study for the side effects (SE) of CoronaVac was carried out in February 2021 among Turkish healthcare workers who were recently vaccinated. The questionnaire inquired about local and systemic SEs that occurred in the short-term, within four weeks, following vaccination. Results: A total of 780 healthcare workers were included in this study; 62.5% of them experienced at least one SE. Injection site pain (41.5%) was the most common local SE, while fatigue (23.6%), headache (18.7%), muscle pain (11.2%) and joint pain (5.9%) were the common systemic SEs. Female healthcare workers (67.9%) were significantly more affected by local and systemic SEs than male colleagues (51.4%). Younger age, previous infection, and compromised health status (chronic illnesses and regular medicines uptake) can be associated with an increased risk of CoronaVac SEs; Conclusions: The independent research shows a higher prevalence of CoronaVac SEs than what is reported by phase I–III clinical trials. In general, the results of this study confirm the overall safety of CoronaVac and suggest potential risk factors for its SEs. Gender-based differences and SEs distribution among age groups are worth further investigation.

## 1. Introduction

Aversion to side effects (SEs) of vaccines, even minor SEs, can increase vaccine hesitancy levels at the time when accelerating mass vaccination is needed the most [1]. A recent cross-sectional study found that the risk of COVID-19 vaccine hesitancy was the highest for Chinese vaccines with 50% efficacy and 1 in 10,000 serious SE [2]. The WHO’s Strategic Advisory Group of Experts on Immunization (SAGE) suggests that mistrust in safety data provided by pharmaceutical companies may play a key role in decreasing vaccine acceptance levels [3,4]. Therefore, independent (non-sponsored) studies for vaccines’ safety and SEs is a crucial asset to enhance public confidence in COVID-19 vaccines and their effectiveness.

CoronaVac (Sinovac Life Sciences, Beijing, China) is an inactivated virus vaccine that was one of the earliest to join the COVID-19 vaccine trials pipeline in April 2020 [5]. Based on its efficacy and safety results of phase I/II trials, the vaccine was subjected to emergency use approvals (EUAS) in a number of countries that considered its economic value. The currently available peer-reviewed evidence of CoronaVac confirms its safety for human use in the short term as well as its efficacy [6,7]. Serious SEs were not recorded in any trial phase I–III, with all the reported SEs being predictable, of minor nature, and limited prevalence [8]. The Turkish government announced three main criteria for selecting the vaccine to be used in mass vaccination; the broadest coverage, availability in the shortest time, and the greatest degree of safety [9]. According to the Turkish phase III trial, CoronaVac showed 91.25% efficacy among vaccinated volunteers; therefore, it acquired EUA in early 2021 [10].

Heretofore, the best available evidence suggests that the innate, humoral, and cell-mediated responses to viral vaccines can differ between females and males [11]. Therefore, the interest in understanding the mechanisms of these differences has grown in recent years, complemented by emerging epidemiologic evidence that supported the existence of females’ increased susceptibility to vaccines’ SEs [11,12].

The primary objective of this study was to estimate the prevalence of CoronaVac SEs in Turkey among healthcare workers. The secondary objective was to evaluate the association between SE levels and demographic and medical variables that may predict risk factors for SEs’ prevalence and intensity.

## 2. Materials and Methods

### 2.1. Design

A cross-sectional survey-based study was carried out in February 2021 to evaluate the prevalence of CoronaVac short-term SEs among healthcare workers in Turkey where the vaccine acquired emergency use authorization, and the healthcare workers were defined as a “priority group” that was planned to be vaccinated since 14 January 2021 [13]. The study used a validated self-administered questionnaire that was developed and disseminated digitally using KoBoToolbox (Harvard Humanitarian Initiative, Cambridge, MA, USA 2021) [14]. The study protocol was registered in the National Library of Medicine (NLM) registry—NCT047706156, and it was conducted and reported according to the STROBE guidelines for cross-sectional studies [15,16].

### 2.2. Participants

Given the nature of this study, which is based on self-assessed and self-reported outcomes, the healthcare workers were selected as the target population due to their comparatively high levels of health literacy and scientific interest. The healthcare workers in the territory of Turkey who received either one or two doses of the CoronaVac vaccine during the last 30 days were included in this study. Meanwhile, non-healthcare workers, e.g., senior adults (+65 years old) and social workers who were vaccinated by CoronaVac were excluded. Participation in this study was voluntary, and the participants received no financial compensation in order to minimize self-selection bias.

### 2.3. Instrument

The questionnaire used in this study consisted of twenty compulsory multiple-choice items and eight conditional items. It had been adapted and socioculturally validated from pre-existing studies on vaccines’ SEs, and its content validity was reviewed by an expert panel. Iterative discussion was used to finalize the questionnaire and its test re-test reliability yielded a mean Cohen’s kappa coefficient of 0.89 ± 0.13 (0.54–1). The first section of the questionnaire covered the demographic information including age, gender, geographic region, specialty, and length of work experience. The second section was about their medical anamneses, while the third one was about COVID-19-related anamnesis. The fourth section explored the short-term local and systemic SEs that emerged after receiving the CoronaVac COVID-19 vaccine (the questionnaire is in Table S1). The validation process and psychometric properties of the questionnaire were previously reported in detail [17].

### 2.4. Data Collection

After ethical clearance, participants were recruited using convenience sampling. Administrative staff of private chains as well as local state-run hospitals were requested to spread the survey in their own centralized cross-platform messaging groups. In order to include dentists working in their own private clinics, the Turkish Dental Association was asked to send the survey to its members through a distribution list.

### 2.5. Ethics

The study protocol was reviewed and approved by the General Directorate of Health Services of the Turkish Ministry of Health within the scope of the Public Health Law no. 1593; additionally, it had been approved by the Ethics Committee of the Faculty of Medicine, Masaryk University (MUNI) on 20 January 2021 (Ref. 2/2021) [18]. All the participants had to provide their informed consent digitally before joining the study, and the data were stored and handled by MUNI in compliance with the General Data Protection Regulation (GDPR) [19].

### 2.6. Analysis

All statistical tests were carried out by the Statistical Package for the Social Sciences (SPSS) version 27.0 (SPSS Inc., Chicago, IL, USA, 2020). Descriptive statistics for demographic, medical anamneses and SEs were presented by frequencies, percentages, and central tendency measures to describe the study sample and prevalence of CoronaVac SEs. Inferential statistics was carried out to evaluate the impact of demographic and medical anamnestic variables of SEs frequency and intensity. The Shapiro–Wilk test, Chi-squared test (*χ*^2^), and ANOVA tests were used with a confidence level of 95% and a significance level (*p*) of ≤ 0.05. Moreover, binary logistic regression was conducted to calculate the odds ratio of SE occurrence adjusted to age, gender, and medical anamneses with a confidence level of 95%.

## 3. Results

### 3.1. Demographic Characteristics

A total of 878 responses were received between 5 February–5 March 2021 from Turkish participants who recently received the CoronaVac vaccine; 15 non-healthcare workers, 82 individuals vaccinated before 14 January, and 1 non-Turkish healthcare worker were excluded. Out of the 780 included participants, the vast majority were females (67.2%), and living in the Istanbul region (62.1%). Their mean age was 36.12 ± 11.58 (20–74) years, and around 95.9% were physicians, dentists, pharmacists, nurses, and midwives. The majority had 1–5 years of work experience (39%), followed by > 20 years (26.8%), 6–10 years (18.6%), and 11–20 years (15.7%) (Table 1).

### 3.2. Medical Anamneses

A total of 130 (16.7%) participants reported suffering from chronic diseases without significant difference between females (16.8%) and males (16.5%). The most common chronic disease was chronic hypertension (4.4%), followed by other endocrine disorders (3.9%) and other cardiovascular disease (2.7%). Across gender, females were significantly more affected by obstructive pulmonary diseases (*χ*^2^ = 5.2; *p* = 0.023) and other endocrine disorders (*χ*^2^ = 5.3; *p* = 0.021) more than their male counterparts, while males were significantly more affected by chronic hypertension (*χ*^2^ = 6.6; *p* = 0.01). The >32 years old group was significantly more affected by other cardiovascular disease (*χ*^2^ = 9.2; *p* = 0.002), chronic hypertension (*χ*^2^ = 33.7; *p* < 0.001), diabetes mellitus (*χ*^2^ = 11.9; *p* = 0.001), other endocrine disorders (*χ*^2^ = 5.8; *p* = 0.016), and rheumatic disorders (*χ*^2^ = 3.9; *p* = 0.047) compared to the ≤32 years old group.

A total of 181 (23.2%) participants reported receiving medications regularly with a significant difference (*χ*^2^ = 4.9; *p* = 0.027) between females (25.6%) and males (18.4%). Antidepressants (5.3%) were the most commonly used drugs followed by thyroid hormone supplement (4.7%), and antihypertensive drugs (4.2%). Females had significantly higher consumption levels of antidepressants (*χ*^2^ = 4.8; *p* = 0.028), antihistamines (*χ*^2^ = 4.4; *p* = 0.035), and thyroid hormone supplements (*χ*^2^ = 13.2; *p* < 0.001), while males had significantly higher consumption levels of antidiabetics (*χ*^2^ = 3.9; *p* = 0.05), antiepileptics (*χ*^2^ = 5.1; *p* = 0.024), and antihypertensive drugs (*χ*^2^ = 5.5; *p* = 0.019). The older group (>32 years old) had significantly higher levels of anticoagulants (*χ*^2^ = 9; *p* = 0.003),antidiabetics (*χ*^2^ = 15.2; *p* < 0.001), antihypertensive drugs (*χ*^2^ = 26.6; *p* < 0.001), and thyroid hormone supplements (*χ*^2^ = 5.7; *p* = 0.017) compared to the younger group (≤32 years old). In general, the older group had significantly higher levels of drug consumption (*χ*^2^ = 28.4; *p* < 0.001) than the younger group (Table 2).

### 3.3. COVID-19-Related Anamneses

About half (48.8%) of the participants received one dose of the vaccine by the time they filled in the questionnaire, with no significant difference (*χ*^2^ = 0.3; *p* = 0.581) between males (50.2%) and females (48.1%). The difference between age groups was statistically significant (*χ*^2^ = 23.6; *p* < 0.001), as 57.2% of the younger group received one dose, while 39.8% of the older group received one dose. The median interval between the first dose and the second dose was 28 days, which corresponds to the recommended 4-week interval by the Turkish Ministry of Health [9]. Regarding their previous COVID-19 infection, 9.8% of the participants reported being infected previously with SARS-CoV-2. There was no significant difference across gender or age groups in terms of previous infection (Table 3).

### 3.4. Prevalence of CoronaVac Side Effects

Out of the 780 included participants, 487 (62.5%) participants reported suffering from at least one SE after receiving the vaccine. In general, females (67.9%) were significantly (*χ*^2^ = 20.1; *p* < 0.001) more affected by SEs compared to males (51.4%). Regarding the local SEs, injection site pain (41.5%) was the most common local SE, followed by injection site swelling (2.6%) and injection site redness (1.4%). Females (47.3%) were significantly (*χ*^2^ = 17; *p* < 0.001) more affected by local SEs than males (31.8%), and the younger group (48.3%) was significantly (*χ*^2^ = 12.4; *p* = 0.001) more affected by local SEs than the older group (35.8%).

**Figure 1:**
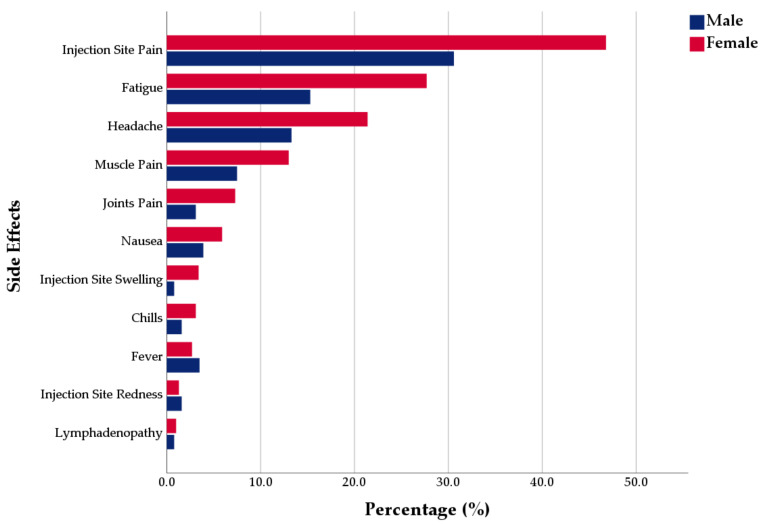
Gender difference of CoronaVac general side effects reported by Turkish healthcare workers, from January–February 2021.

Regarding the systemic SEs, fatigue (23.6%) was the most common systemic SE, followed by headache (18.7%), muscle pain (11.2%), joint pain (5.9%), and nausea (5.3%). Females were reportedly more affected by all systemic SEs, except fever, which was slightly more common in males. Fatigue, headache, muscle pain, and joint pain were significantly more common in females (27.7%, 21.4%, 13%, 7.3%, respectively) than males (15.3%, 13.3%, 7.5%, 3.1%, respectively). The younger group was insignificantly (*U* = 71990; *p* = 0.171) more affected by the systemic SEs than the older group (Figure 1).

Fever (3%), chills (2.6%), and lymphadenopathy (0.9%) were not commonly reported by the participants. The majority of SEs were resolved within one day (49.3%) and within three days (36%). The difference of duration was not significantly correlated either for gender, age group, previous infection, or medical treatment. All 23 participants who reported long-standing SEs (over one week) were females with a median age of 30 years, and their SEs were mainly muscle pain (39.1%), headache (39.1%), injection site pain (39.1%), and fatigue (30.4%).

A total of 88 (11.3%) participants reported various forms of oral SEs. The most common oral SEs were bleeding or sore gingiva (4.7%) followed by ulcers, vesicles, or blisters (UVB) (4.4%), and halitosis (2.1%). However, taste disorders and tongue-tingling “paresthesia” were unsolicited SEs; 0.9% reported having taste disorders, especially metallic taste, and 0.3% reported tongue paresthesia. Tonsillitis and sore throat were also reported by very few cases. The most common sites of oral mucocutaneous SEs were labial and buccal mucosa (50%), lips (30%), and gingiva (26.7). The majority (63.6%) of oral SEs emerged within the first week after vaccination. A total of 18 (2.3%) participants reported having skin-related SEs, which were mainly skin rash (1.5%) and urticaria (0.8%). Females were also significantly (*χ*^2^ = 5.9; *p* = 0.015) more affected by skin rash. The most common locations of skin-related SEs were chest and trunk (42.1%) and face (36.8%). Regarding the severe SEs, only four participants (0.5%) reported severe SEs that required medical care, all of them were females (100%) and they were equally distributed among both age groups (≤32 years old vs. >32 years old). Three were physicians from the Istanbul region, and one was a nurse from the West Marmara region (Table 4).

### 3.5. Risk Factors of CoronaVac Side Effects

The intensity of CoronaVac SEs was depicted by the mean of total SEs experienced by an individual, which ranged between (0–11). Females (1.33 ± 1.36) significantly had (*U* = 51480; *p* < 0.001) a higher level of SE intensity than their male colleagues (0.82 ± 1.16). Age was also a significant demographic risk factor for SE prevalence and intensity. Using the median population age (32 years) as a cut-off for participants’ age, the younger group had significantly higher prevalence (*χ^2^* = 6.6; *p* = 0.01) and intensity (*U* = 67938; *p* = 0.007) of general SEs than the older group. Using the retirement age of the Turkish population (60 years) as a cut-off for participants’ age, the younger group had a significantly higher prevalence (*χ*^2^ = 16.4; *p* < 0.001) and intensity (*U* = 8767; *p* < 0.001) of general SEs than the older group.

Chronic illnesses were insignificantly associated with an increased risk of SEs emergence and intensity. Medical treatments were significantly associated with an increased prevalence (*χ*^2^ = 6; *p* = 0.014) and intensity (*U* = 47897; *p* = 0.013) of general SEs. The participants who received two doses (63.4%) had a slightly higher level of SEs compared to those who received one dose (61.4%). The prevalence of SEs was significantly higher in the previously infected participants (*χ^2^* = 3.9; *p* = 0.05), while the SE intensity was not significantly different (Table 5).

The binary logistic regression revealed that after adjusting age and medical anamneses (chronic illness and medical treatment), females had an adjusted odds ratio (AOR) to experience SEs following CoronaVac 1.837 times (CI 95%: 1.344–2.511) higher than their male counterparts. The adjustment of gender and medical anamneses revealed that the younger age group (≤32 years old) had an AOR to experience SEs 1.566 times (CI 95%: 1.155–2.124) higher than the older age group (>32 years old). Additionally, age and gender adjustment revealed that people with chronic illnesses had an AOR 1.090 times (CI 95%: 0.645–1.842) higher than their counterparts without chronic illnesses; also, people taking regular medications had an AOR 1.797 times (CI 95%: 1.117–2.891) higher than those who do not take medications regularly.

## 4. Discussion

On 13 January 2021, CoronaVac acquired EUA from the Turkish Medicines and Medical Devices Agency (TMMDA) based on the initial efficacy (91.25%) results of a nationwide phase III trial led by researchers of Hacettepe University (Ankara, Turkey) [8]. As of 1 April 2021, CoronaVac had acquired EUA in 18 countries besides its manufacturer’s country (China), with over 70 million doses administered globally [20]. However, seven phase III trials for the safety and effectiveness of CoronaVac were registered in Brazil, Chile, China, Hong Kong, Indonesia, and Turkey; there is still a lack of peer-reviewed evidence on its SEs [21].

According to the evaluation report of the Food and Health Bureau (FHB) of Hong Kong SAR on CoronaVac, the “very common” adverse reactions (≥10%) were injection site pain, headache, and fatigue [22]. Our results support this claim, as injection site pain (41.5%), fatigue (23.6%), and headache (18.7%) were reported by more than 10% of the participants. While the FHB report described muscle pain as a “common” adverse reaction (1–10%), it was slightly more prevalent in our sample (11.2%). Injection site swelling and injection site redness were common local adverse reactions according to the FHB report, and they were reported by 2.6% and 1.4%, respectively of our participants. Nausea, chills, and joint pain were common systemic adverse reactions according to the FHB report, and they were reported by 5.3%, 2.6%, and 5.9%, respectively, of our participants [22]. However, fever was described as an “uncommon” adverse reaction (0.1–1%); it was reported by 3% of our participants. Similarly, skin rashes (1.5%) were more prevalent in our study, while all skin-related adverse reactions were depicted as “uncommon” by the FHB. Skin flushing, urticaria, and acne were experienced by 0.1, 0.8%, and 0.1%, respectively, of our participants, and they were found to be “uncommon” by the FHB. Hyposmia was a “rare” adverse reaction (<0.01%) according to the FHB. This could be challenged by the unpredicted higher level of taste disorders reported by 0.9% of our participants, even though these disorders were unsolicited SEs [22].

The currently available peer-reviewed evidence on CoronaVac safety is from phase I/II trials which were carried out in the manufacturer’s country [6,7]. However, these trials employed various concentration doses with different intervals between first and second dose, the 3 μg dose with 28-days interval was recommended for phase III trials and subsequent phases. In the phase I/II phase trials, injection site pain was the most prevalent local SE among the ≤59 years old group (10.4%) and the >59 years old group (11%). Our independent study also found that injection site pain was the most prevalent local SE; however, the difference between the ≤59 years old group (42.7%) and the >59 years old group (13.9%) was statistically significant (*χ*^2^ = 11.8; *p* = 0.001). While injection site redness was not recorded in the 3 μg group, it was reported in the 6 μg group by 0.7% of the ≤59 years old group and 1% of the >59 years old group [6,7]. This slight difference between the two age groups was also noticed in our sample: 1.3% vs. 2.8%, respectively. Fatigue was the most common systemic SE in both the phase I/II trials and our study, and the ≤59 years old group was more affected (6.9% and 24.2%, respectively) than the >59 years old group (3% and 11.1%, respectively) [6,7].

In the ≤59 years old group, the prevalence of systemic SEs was higher in our study than the phase I/II trials. Headache, muscle pain, and nausea were experienced by 19%, 11.6% and 5.2% of our sample, while only 2.1%, 1.4%, and 1.4% of the phase I/II participants experienced them. On the other hand, fever had a relatively low prevalence in both the phase I/II trials and our study: 2.8% and 3%, respectively [6]. Both local and systemic SEs were more prevalent after the first dose (8.3% and 10.4%, respectively) than the second dose (2.8% and 3.6%, respectively) [6]. Similarly, the prevalence of systemic SEs was also higher for the >59 years old group. Headache was not experienced by any of the 3 μg group participants, while five (4%) participants of the 6 μg group reported it [7]. Muscle pain and nausea were experienced by 2.8% and 5.6% of our participants and 2% and 1% of the phase I/II participants [7]. Local SEs were more prevalent after the second dose (7.3%) than the first dose (6.5%). Contrarily, systemic SEs were much less prevalent after the second dose (0.8%) than the first dose (9.7%) [7].

The overall prevalence of CoronaVac SEs in this independent study (local: 42.2% and systemic: 40.6%) is significantly lower than mRNA vaccines SEs reported by the Centers for Disease Control and Prevention (CDC) based on phase III trials, Pfizer–BioNTech (local: 84.7% and systemic: 77.4%) and Moderna (local: 86% and systemic: 66.6%) [23,24]. This is also supported by the findings of our independent study of Pfizer–BioNTech COVID-19 vaccine SEs, which showed that 93.1% of all vaccinated healthcare workers in the Czech Republic experienced at least one SE either locally or systemically [17]. Moreover, the SE prevalence of viral vector vaccine of AstraZeneca–Oxford reported by the European Medicines Agency (EMA) was considerably higher than CoronaVac [25]. These findings should be cautiously interpreted in relation to vaccine efficacy reports as CoronaVac has significantly heterogeneous efficacy data ranging between 50.4% to 91.25% [8].

The differences in SE prevalence of our independent study from the phase I/II trials should be interpreted in light of the limited sample size of phase I/II trials and their restricted nature for healthy participants. Gender was the most influential factor for SE emergence and intensity according to our sample, even though the safety data of the phase I/II trials were not stratified by gender. While our non-random sampling technique yielded a national representative sample for the healthcare worker population in Turkey, where females represent 60% of the total health workforce, the phase I/II trials recruited a gender-balanced sample (1:1) [26,27].

The gender-based differences in terms of vaccines’ SEs had been consistently reported in several viral vaccines in the past, including influenza, measles-mumps-rubella combination vaccine (MMR), attenuated Japanese encephalitis, and attenuated Dengue vaccines [11,12]. These differences are usually not in favor of females, as they develop stronger immune responses and more frequent and more intense SEs. Various hypotheses were proposed to explain these differences, including adaptive immunity-related theories, sex steroid-related theory, and innate immunity-related theories (pattern recognition receptors PRRs, induction of type I IFN responses, and putative androgen and estrogen response elements) [11]. The findings of this study warrant further investigation for the gender-related differences of CoronaVac SEs.

This study provides the first evidence on oral SE following CoronaVac administration including oral ulcerations (4.4%), dysgeusia (0.9%), and oral paresthesia (0.3%). However, oral SEs were rarely reported, and some of these oral mucocutaneous lesions and oral conditions were previously reported by COVID-19 patients [28,29,30,31,32,33,34].

### 4.1. Limitations

This study is naturally limited by its cross-sectional design that relies on self-assessed and self-reported outcomes; therefore, healthcare workers were targeted as they retain high levels of health literacy and scientific interests. Additionally, some SEs were not solicited in this study while they had been questioned in the phase I/II trials, e.g., diarrhea, and cough, etc. The non-random snowballing technique was used for data collection, although we have obtained the representativeness of our data for age and gender for Turkish healthcare workers.

### 4.2. Implications

1. Further independent studies for CoronaVac effectiveness and SE are critically required to be carried out by academic institutions.
2. Healthcare workers and students represent an ideal population group to take part in active surveillance studies due to their high level of health literacy and scientific interest.
3. The upcoming studies of CoronaVac SE should explore the gender-based differences as well as the role of medical anamneses on SE prevalence and intensity.

## 5. Conclusions

This study provides the first independent evidence on the prevalence of CoronaVac SEs, which was found to be 62.5%. Injection site pain (41.5%) was the most common local SE, while fatigue (23.6%), headache (18.7%), muscle pain (11.2%) and joint pain (5.9%) were the common systemic SEs. Female healthcare workers (67.9%) were significantly more affected by both local and systemic SEs compared to their male counterparts (51.4%). Younger age, previous infection, and compromised health status (chronic illnesses and regular medicines uptake) can be associated with an increased risk of CoronaVac SEs. Most SEs are rather minor reactions that persist in most cases for one to three days. Further studies are required to explore the gender-based differences as well as the prevalence of CoronaVac SEs in other population groups.

## Figures and Tables

**Table 1:** Demographic characteristics of Turkish healthcare workers vaccinated by CoronaVac, from January–February 2021.

Variable	Outcome	Frequency	Percentage
Gender	Female	524	67.2%
	Male	255	32.7%
	Non-binary	1	0.1%
Age	≤32 years old	403	51.7%
	>32 years old	377	48.3%
Profession	Physician	250	32.1%
	Dentist	295	37.8%
	Pharmacist	150	19.2%
	Nurse	43	5.5%
	Midwife	10	1.3%
	Physiotherapist	6	0.8%
	Lab Worker	25	3.2%
	Dietitian	1	0.1%
Experience	1–5 years	298	39%
	6–10 years	142	18.6%
	11–20 years	120	15.7%
	>20 years	205	26.8%
Region (NUTS-1)*	Istanbul (TR1)	484	62.1%
	West Marmara (TR2)	64	8.2%
	Aegean (TR3)	44	5.6%
	East Marmara (TR4)	33	4.2%
	West Anatolia (TR5)	55	7.1%
	Mediterranean (TR6)	26	3.3%
	Central Anatolia (TR7)	14	1.8%
	West Black Sea (TR8)	18	2.3%
	East Black Sea (TR9)	10	1.3%
	Northeast Anatolia (TRA)	2	0.3%
	Central East Anatolia (TRB)	14	1.8%
	Southeast Anatolia (TRC)	16	2.1%

* NUTS-1: the first-level of Nomenclature of Territorial Units for Statistics system.

**Table 2:** Medical anamneses of Turkish healthcare workers vaccinated by CoronaVac, from January–February 2021.

Variable	Outcome	Female	Male	Total	*Sig.*
Chronic Illness	Blood Disease	0 (0%)	2 (0.8%)	2 (0.3%)	0.107 *
	Cancer	3 (0.6%)	2 (0.8%)	5 (0.6%)	0.664 *
	Chronic Hypertension	16 (3.1%)	18 (7.1%)	34 (4.4%)	0.010
	Other Cardiovascular Disease	15 (2.9%)	6 (2.4%)	21 (2.7%)	0.680
	Dermatologic Disease	2 (0.4%)	0 (0%)	2 (0.3%)	1.000 *
	Diabetes Mellitus	6 (1.1%)	5 (2%)	11 (1.4%)	0.353 *
	Other Endocrine Disorders	26 (5%)	4 (1.6%)	30 (3.9%)	0.021
	Gastrointestinal Disorders	7 (1.3%)	3 (1.2%)	10 (1.3%)	1.000 *
	Liver Disease	0 (0%)	2 (0.8%)	2 (0.3%)	0.107 *
	Obstructive Pulmonary Diseases	15 (2.9%)	1 (0.4%)	16 (2.1%)	0.023
	Ophthalmologic Disease	1 (0.2%)	0 (0%)	1 (0.1%)	1.000 *
	Psychologic Distress	0 (0%)	2 (0.8%)	2 (0.3%)	0.107 *
	Neurologic Disease	4 (0.8%)	4 (1.6%)	8 (1%)	0.449 *
	Rheumatic Disorders	6 (1.1%)	1 (0.4%)	7 (0.9%)	0.437 *
	Total	88 (16.8%)	42 (16.5%)	130 (16.7%)	0.910
Medical Treatment	Antiarrhythmic	8 (1.5%)	3 (1.2%)	11 (1.4%)	1.000 *
	Anti-asthmatic	5 (1%)	0 (0%)	5 (0.6%)	0.179 *
	Antibiotics	1 (0.2%)	1 (0.4%)	2 (0.3%)	0.548 *
	Anticoagulants	8 (1.5%)	7 (2.7%)	15 (1.9%)	0.271 *
	Antidepressants	34 (6.5%)	7 (2.7%)	41 (5.3%)	0.028
	Antidiabetics	6 (1.1%)	8 (3.1%)	14 (1.8%)	0.080 *
	Antiepileptics	1 (0.2%)	4 (1.6%)	5 (0.6%)	0.042 *
	Antihistamines	9 (1.7%)	0 (0%)	9 (1.2%)	0.035 *
	Antihypertensive	16 (3.1%)	17 (6.7%)	33 (4.2%)	0.019
	Cholesterol Medications	2 (0.4%)	6 (2.4%)	8 (1%)	0.017 *
	Oral Contraceptives	4 (0.8%)	-	-	-
	Gastrointestinal Medications	4 (0.8%)	3 (1.2%)	7 (0.9%)	0.689 *
	Immunosuppressives	6 (1.1%)	3 (1.2%)	9 (1.2%)	1.000 *
	Thyroid Hormone Supplement	35 (6.7%)	2 (0.8%)	37 (4.7%)	<0.001
	Other Drugs	14 (2.7%)	4 (1.6%)	18 (2.3%)	0.336
	Total	134 (25.6%)	47 (18.4%)	181 (23.2%)	0.027

Chi-squared test and Fisher’s exact test (*) were used with a significance level (*Sig.*) of ≤0.05.

**Table 3:** COVID-19-related anamneses of Turkish healthcare workers vaccinated by CoronaVac, from January–February 2021.

Variable	Outcome	Female	Male	Total	*Sig.*
Doses	One	252 (48.1%)	128 (50.2%)	380 (48.8%)	0.581
	Two	272 (51.9%)	127 (49.8%)	399 (51.2%)	
Interval	days	28.01 ± 3.02	27.50 ± 3.34	27.85 ± 3.13	0.741
Infection	Yes	52 (9.9%)	24 (9.4%)	76 (9.8%)	0.821

Chi-squared test and ANOVA test were used with a significance level (*Sig.*) of ≤0.05.

**Table 4:** Prevalence of CoronaVac side effects reported by Turkish healthcare workers, from January–February 2021.

Variable	Outcome	Female	Male	Total	*Sig*.
Local SE	Injection Site Pain	245 (46.8%)	78 (30.6%)	323 (41.5%)	<0.001
	Injection Site Swelling	18 (3.4%)	2 (0.8%)	20 (2.6%)	0.028
	Injection Site Redness	7 (1.3%)	4 (1.6%)	11 (1.4%)	0.756 *
	Total (μ ± SD)	0.52 ± 0.58	0.33 ± 0.49	0.45 ± 0.56	<0.001
Systemic SE	Fatigue	145 (27.7%)	39 (15.3%)	184 (23.6%)	<0.001
	Headache	112 (21.4%)	34 (13.3%)	146 (18.7%)	0.007
	Nausea	31 (5.9%)	10 (3.9%)	41 (5.3%)	0.242
	Muscle Pain	68 (13%)	19 (7.5%)	87 (11.2%)	0.022
	Chills	16 (3.1%)	4 (1.6%)	20 (2.6%)	0.2190
	Joint Pain	38 (7.3%)	8 (3.1%)	46 (5.9%)	0.022
	Fever	14 (2.7%)	9 (3.5%)	23 (3%)	0.507
	Lymphadenopathy	5 (1%)	2 (0.8%)	7 (0.9%)	1.000 *
	Total (μ ± SD)	0.82 ± 1.15	0.49 ± 0.91	0.71 ± 1.09	<0.001
General SE Duration	1 day	169 (47.9%)	69 (53.1%)	238 (49.3%)	0.311
	3 days	131 (37.1%)	43 (33.1%)	174 (36%)	0.413
	5 days	16 (4.5%)	11 (8.5%)	27 (5.6%)	0.096
	1 week	14 (4%)	7 (5.4%)	21 (4.3%)	0.498
	> 1 week	21 (5.9%)	0 (0%)	21 (4.3%)	0.004
	> 1 month	2 (0.6%)	0 (0%)	2 (0.4%)	1.000 *
Oral SE	Ulcers, Vesicles or Blisters (UVB)	27 (5.2%)	7 (2.7%)	34 (4.4%)	0.123
	Bleeding or Sore Gingiva	27 (5.2%)	10 (3.9%)	37 (4.7%)	0.448
	Halitosis	12 (2.3%)	4 (1.6%)	16 (2.1%)	0.505
	Swollen or Injured Lips	5 (1%)	0 (0%)	5 (0.6%)	0.179 *
	Taste Disorder	6 (1.1%)	1 (0.4%)	7 (0.9%)	0.437 *
	Tongue Tingling	2 (0.4%)	0 (0%)	2 (0.3%)	1.000 *
	White or Red Plaque (WRP)	4 (0.8%)	2 (0.8%)	6 (0.8%)	1.000 *
	Xerostomia	2 (0.4%)	1 (0.4%)	3 (0.4%)	1.000 *
	Total	67 (12.8%)	21 (8.2%)	88 (11.3%)	0.060
UVB-WRP Location	Gingiva	7 (30.4%)	1 (14.3%)	8 (26.7%)	0.638 *
	Labial or Buccal Mucosa	12 (46.2%)	5 (62.5%)	17 (50%)	0.688 *
	Lips	4 (17.4%)	5 (71.4%)	9 (30%)	0.014 *
	Palate	3 (13%)	0 (0%)	3 (10%)	1.000 *
	Tongue	6 (26.1%)	0 (0%)	6 (20%)	0.290 *
Oral SE Onset	1–3 days	30 (44.8%)	7 (33.3%)	37 (42%)	0.354
	1st week	14 (20.9%)	5 (23.8%)	19 (21.6%)	0.768 *
	2nd week	7 (10.4%)	7 (33.3%)	14 (15.9%)	0.035 *
	3rd week	10 (14.9%)	1 (4.8%)	11 (12.5%)	0.448 *
	4th week	6 (9%)	1 (4.8%)	7 (8%)	1.000 *
Skin-related SE	Acne	1 (0.2%)	0 (0%)	1 (0.1%)	1.000 *
	Rash	12 (2.3%)	0 (0%)	12 (1.5%)	0.011 *
	Urticaria	5 (1%)	1 (0.4%)	6 (0.8%)	0.670 *
	Total	17 (3.2%)	1 (0.4%)	18 (2.3%)	0.013
Skin-related SE Location	Face	6 (35.3%)	1 (50%)	7 (36.8%)	1.000 *
	Upper Limb	2 (11.8%)	0 (0%)	2 (10.5%)	1.000 *
	Lower Limb	1 (5.9%)	0 (0%)	1 (5.3%)	1.000 *
	Chest or Trunk	8 (47.1%)	0 (0%)	8 (42.1%)	0.485 *
	Back	1 (5.9%)	0 (0%)	1 (5.3%)	1.000 *
Severe SE	Total	4 (0.8%)	0 (0%)	4 (0.5%)	0.309 *

Chi-squared test and Fisher’s exact test (*), and ANOVA test were used with a significance level (*Sig.*) of ≤0.05; SE = Side Effects; SD = Standard Deviation; UVB-WRP = Ulcers, vesicles, blisters, and white and red plaque.

**Table 5:** Risk factors of CoronaVac general side effects reported by Turkish healthcare workers, from January–February 2021.

Variable	Outcome	SEs Prevalence		SEs Intensity	
		N (%)	Sig.	μ ± SD	Sig.
Gender	Female	356 (67.9%)	<0.001	1.33 ± 1.36	<0.001
	Male	131 (51.4%)		0.82 ± 1.16	
	Non-binary	0 (%)		0	
Age	≤32 years old	269 (66.7%)	0.010	1.27 ± 1.31	0.007
	>32 years old	218 (57.8%)		1.06 ± 1.29	
	≤59 years old	476 (64%)	<0.001	1.19 ± 1.28	<0.001
	>59 years old	11 (30.6%)		0.64 ± 1.59	
Chronic Illness	No	400 (61.5%)	0.247	1.13 ± 1.27	0.135
	Yes	87 (66.9%)		1.33 ± 1.46	
Medical Treatment	No	360 (60.1%)	0.014	1.12 ± 1.32	0.013
	Yes	127 (70.2%)		1.31 ± 1.26	
Previous Infection	No	431 (61.3%)	0.050	1.16 ± 1.33	0.218
	Yes	56 (72.7%)		1.21 ± 1.07	
Doses	One	234 (61.4%)	0.566	1.13 ± 1.31	0.446
	Two	253 (63.4%)		1.20 ± 1.30	

Chi-squared test and Mann–Whitney tests were used with a significance level (*Sig.*) of ≤0.05; SE = Side Effects; SD = Standard Deviation.

## Data Availability

The data that support the findings of this study are available from the corresponding author upon reasonable request.

## Supplementary Materials

The following are available online at https://www.mdpi.com/article/10.3390/jcm10122629/s1, Table S1: OSECV Instrument (in Turkish).
