# Supplementary material for: Prevalence and Risk Factors of CoronaVac Side Effects: An Independent Cross-Sectional Study among Healthcare Workers in Turkey
Source: J Clin Med. 2021 Jun 15;10(12):2629. doi: 10.3390/jcm10122629 (PMC8232682; doi:10.3390/jcm10122629)
Supplement: Supplementary file 1 [file jcm-10-02629-s001.zip › jcm-1209485-supplementary.pdf]

Journal of
Clinical Medicine

MDPI

Supplementary Material

Table S1. OSECV Instrument (in Turkish).

**1. Demographic Data**	
**I. Gender**	o Female<br>o Male<br>o Non-binary<br>o Prefer not to say
**II. Age**	o Dropdown menu with numbers (18-99)
**III. Profession**	o Physician (MD)<br>o Dentist (DDS)<br>o Nurse (BSN)<br>o Midwife<br>o Physiotherapist<br>o If other *(please specify)*
**In the case of "Physician (MD)", select your speciality**	o Allergology<br>o Anaesthetics<br>o Cardiac surgery<br>o Cardiology<br>o Cardo-thoracic surgery<br>o Chemical pathology<br>o Child and adolescent psychiatry<br>o Clinical oncology<br>o Clinical pharmacology and therapeutics<br>o Clinical radiology<br>o Dermato-venereology<br>o Emergency medicine<br>o Endocrinology<br>o Gastroenterology<br>o General (internal) medicine<br>o General psychiatry<br>o General surgery<br>o Geriatric medicine<br>o Haematology<br>o Histopathology<br>o Immunology<br>o Infectious diseases<br>o Maxillo-facial surgery<br>o Medical microbiology and virology<br>o Nephrology<br>o Neurology<br>o Neuropsychiatry

	○ Neurosurgery<br>○ Nuclear medicine (Radiation Oncology)<br>○ Obstetrics and gynecology<br>○ Occupational medicine<br>○ Ophthalmology<br>○ Orthopedics<br>○ Otolaryngology<br>○ Pediatric surgery<br>○ Pediatrics<br>○ Physiotherapy<br>○ Plastic surgery<br>○ Public health medicine<br>○ Respiratory medicine<br>○ Rheumatology<br>○ Urology<br>○ Vascular surgery<br>○ If other *(please specify)*
**IV. City**	○ Istanbul<br>○ Ankara<br>○ Izmir<br>○ If other (please specify)
**V. Working Experience**	○ 1 - 5 years<br>○ 6 - 10 years<br>○ 11 - 20 years<br>○ > 20 years
**2. Medical Anamnesis**	
**VI. Do you have any chronic disease?**	○ Yes<br>○ No
**If "Yes", please select at least one option from the following**	○ Asthma<br>○ Blood Disorder<br>○ Bone Disease<br>○ Bowel Disease<br>○ Cancer<br>○ Cardiac Disease<br>○ Chronic Hypertension<br>○ Chronic Obstructive Pulmonary Disease (COPD)<br>○ Diabetes Mellitus<br>○ Renal Disease<br>○ Rheumatoid Arthritis<br>○ If other *(please specify)*
**VII. Do you take any medication currently?**	○ Yes<br>○ No

**VIII. If "Yes", please select at least one option from the following**	○ Antibiotics<br>○ Antidepressants<br>○ Antihistamine<br>○ Immunosuppressive<br>○ Narcotic pain medications<br>○ Seizure medications<br>○ If other *(please specify)*
**3. COVID-19-related Anamnesis**	
**IX. Vaccination Date (first dose)**	○ Please select the date from the (Calendar)
**X. Have you taken the second dose?**	○ Yes<br>○ No
**XI. Vaccine Type**	Pfizer-BioNTech COVID-19 Vaccine<br>Moderna COVID-19 Vaccine<br>Oxford–AstraZeneca COVID-19 Vaccine<br>Sinovac Vaccine<br>Janssen Vaccine<br>Sputnik Vaccine
**XII. Have you ever been diagnosed with COVID-19?**	○ Yes<br>○ No
**If "Yes", please specify the day when you recovered**	○ Please select the date from the (Calendar)
**XIII. Have you been exposed to PCR-confirmed COVID-19 cases?**	○ Yes<br>○ No
**4. Vaccine Side Effects**	
**XIV. Within four weeks of receiving the vaccine, have you suffered from any of the following oral symptoms?**	○ Ulcers<br>○ Vesicles<br>○ Blisters<br>○ A white or red plaque<br>○ Unpleasant mouth odour<br>○ Bleeding gum<br>○ Burning gingiva<br>○ Swelling of the lips<br>○ If other *(please specify)*
**If "Ulcers", "Vesicles" or "blisters", please choose one location at least**	○ Tongue<br>○ Palate<br>○ Labial or buccal mucosa<br>○ Gingiva<br>○ Lips
**If "White or red plaque", please choose on location at least**	○ Tongue dorsum<br>○ Soft palate<br>○ Labial or buccal mucosa
**XV. When did the oral symptoms emerge?**	○ 1-3 days after vaccination

	○ During 1st week of vaccination<br>○ During 2nd week of vaccination<br>○ During 3rd week of vaccination<br>○ During 4th week of vaccination
**XVI. Within four weeks of receiving the vaccine, have you suffered from any of the following symptoms?**	Injection site pain<br>Injection site swelling<br>Injection site redness<br>Tiredness<br>Headache<br>Nausea<br>Feeling unwell<br>Muscle Pain<br>Chills<br>Joint Pain<br>Fever<br>Swollen lymph nodes (lymphadenopathy)<br>None
**XVII. If you chose any of the previous symptoms, please indicate their duration**	○ 1 day<br>○ 3 days<br>○ 5 days<br>○ 1 week<br>○ > 1 week<br>○ > 1 month
**XVIII. Within four weeks of receiving the vaccine, have you suffered from any severe symptom that required hospitalization, e.g., difficulty in breathing, swelling of face or throat, fast heartbeat, fever, or generalized rash?**	○ Yes<br>○ No
**XIX. Within four weeks of receiving the vaccine, have you suffered from any of the following cutaneous symptoms?**	○ Rash<br>○ Urticaria<br>○ Angioedema<br>○ If other *(please specify)*
**In the case of "Rash", "Urticaria", or "Angioedema", please select the affected site**	Face<br>Upper limb<br>Lower limb<br>Chest / trunk<br>Back
